# Establishment of Motor Neuron-V3 Interneuron Progenitor Domain Boundary in Ventral Spinal Cord Requires Groucho-Mediated Transcriptional Corepression

**DOI:** 10.1371/journal.pone.0031176

**Published:** 2012-02-17

**Authors:** Keith J. Todd, Nathalie Lan-Chow-Wing, Adele Salin-Cantegrel, Anthony Cotter, Chrissandra J. Zagami, Rita Lo, Stefano Stifani

**Affiliations:** Montreal Neurological Institute, McGill University, Montreal, Quebec, Canada; Universidade Federal do Rio de Janeiro, Brazil

## Abstract

**Background:**

Dorsoventral patterning of the developing spinal cord is important for the correct generation of spinal neuronal types. This process relies in part on cross-repressive interactions between specific transcription factors whose expression is regulated by Sonic hedgehog. Groucho/transducin-like Enhancer of split (TLE) proteins are transcriptional corepressors suggested to be recruited by at least certain Sonic hedgehog-controlled transcription factors to mediate the formation of spatially distinct progenitor domains within the ventral spinal cord. The aim of this study was to characterize the involvement of TLE in mechanisms regulating the establishment of the boundary between the most ventral spinal cord progenitor domains, termed pMN and p3. Because the pMN domain gives rise to somatic motor neurons while the p3 domain generates V3 interneurons, we also examined the involvement of TLE in the acquisition of these neuronal fates.

**Methodology and Principal Findings:**

A combination of *in vivo* loss- and gain-of-function studies in the developing chick spinal cord was performed to characterize the role of TLE in ventral progenitor domain formation. It is shown here that TLE overexpression causes increased numbers of p3 progenitors and promotes the V3 interneuron fate while suppressing the motor neuron fate. Conversely, dominant-inhibition of TLE increases the numbers of pMN progenitors and postmitotic motor neurons.

**Conclusion:**

Based on these results, we propose that TLE is important to promote the formation of the p3 domain and subsequent generation of V3 interneurons.

## Introduction

Patterning of the vertebrate central nervous system (CNS) results in the generation of specific neural cell types in precise regions of the CNS. In the developing spinal cord, dorsoventral patterning is regulated by the release of morphogens that form concentration gradients. Specifically, Sonic Hedgehog (Shh) released from the notochord and floor plate is essential for ventral spinal cord patterning [1], [2]. Shh controls the expression of particular homeodomain (HD) and basic helix-loop-helix (bHLH) transcription factors in the ventral spinal cord in a concentration-dependent manner. Some transcription factors (‘Class I’, including the HD proteins Pax6, Dbx1, Dbx2) are repressed by Shh and are therefore found more dorsally, while others (‘Class II, including the HD proteins Nkx2.2 and Nkx6.1) are activated by Shh and are expressed more ventrally.

**Figure 1:**
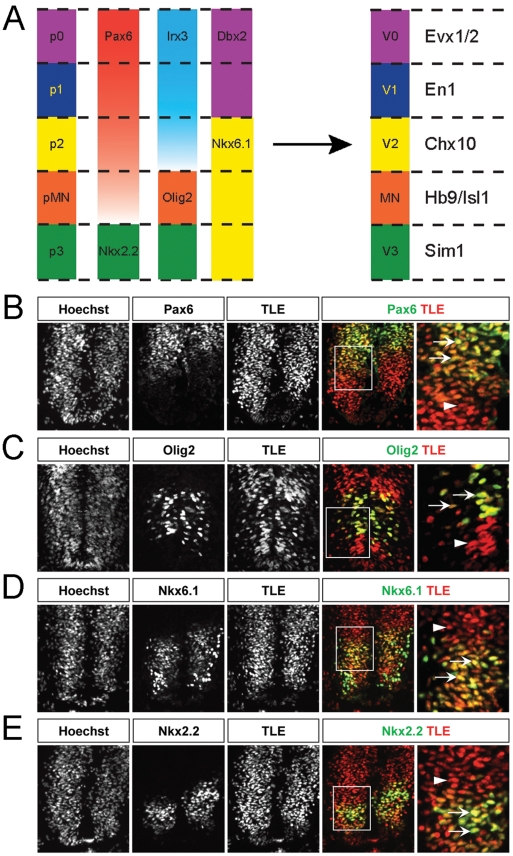
TLE expression in the embryonic chick spinal cord. (A) Schematic representation of the five progenitor cell (p) domains of the ventral spinal cord, termed p0, p1, p2, pMN and p3 from dorsal to ventral positions, respectively. These domains are defined by the specific expression of combinations of HD and bHLH transcription factors. Refinement and maintenance of these progenitor domains is achieved through cross-repressive interactions between pairs of transcription factors, for example between Pax6 and Nkx2.2 at the pMN/p3 boundary. In turn, each progenitor domain generates different neuronal populations, V0, V1 and V2 INs, somatic MNs and V3 INs, respectively. Like the progenitor domains, separate populations of postmitotic neurons can be defined by the expression of specific transcription factors, such as HB9 and Isl1 in MNs derived from the pMN domain or other factors in other cell types, as indicated in the right-hand column. (B–E) Sections through the spinal cord of HH stage 18 chick embryos were subjected to double-labeling immunohistochemical analysis using a panTLE antibody together with antibodies against the indicated proteins. Panels in the right-hand column show high-magnification views of the boxed areas in the adjacent panels. Arrows point to examples of double-labeled cells. Arrowheads point to examples of cells expressing only TLE. TLE expression was observed in most ventral spinal cord cells, including domains p0–p2 (region of high Pax6 immunoreactivity dorsal to the Olig2+ domain), pMN (region expressing Nkx6.1, Olig2 and low levels of Pax6) and p3 (region expressing Nkx6.1 and Nkx2.2) of the ventral area. Notice in particular how virtually all Nkx2.2+ cells also express TLE.

The transduction of the Shh concentration gradient into a differential expression of separate Class I and Class II HD and bHLH transcription factors throughout the ventral spinal cord results in the appearance of separate groups of neural progenitor cells expressing different combinations of Shh-regulated proteins. Specific pairs of Class I and Class II proteins with abutting ventral and dorsal expression domains have the ability to repress each other's expression. As a consequence, different Shh-regulated transcription factors become segregated to defined domains in the ventral spinal cord, so that only one transcription factor of a given cross-repressive pair is expressed in a particular progenitor domain [3], [4]. These combined events result in the establishment of five distinct ventral spinal cord neural progenitor domains characterized by the expression of different combinations of Class I and Class II transcription factors [1]–[4]. Ultimately, the precise combination of transcription factors expressed in each progenitor domain directs the generation of specific neuronal populations during neurogenesis [5]–[7] (Fig. 1A).

One pair of cross-repressive transcription factors in the ventral spinal cord includes the Class II HD protein Nkx2.2 ventrally and the Class I HD protein Pax6 dorsal to the latter. Through mutual transcriptional repression, these proteins contribute to the formation of the boundary between the most ventral progenitor domain, p3, which gives rise to ventral V3 interneurons (INs), and the pMN domain, which generates motor neurons (MNs) [1]–[7].

The mechanisms underlying transcriptional cross-repression between Nkx2.2 and Pax6, as well as between other pairs of Class I and Class II transcription factors, are not well defined. In this regard, several HD transcription factors important for ventral spinal cord patterning, including Nkx2.2, were shown to form complexes with members of the Groucho (Gro)/transducin-like Enhancer of split (TLE) family of transcriptional corepressors [8]–[11]. Vertebrate *Gro/TLE* genes (hereafter collectively referred to as *TLE* for sake of clarity) encode broadly expressed proteins that are activated in the developing spinal cord during ventral patterning [10]. TLE have no intrinsic DNA-binding ability. They rely on interactions with DNA-binding proteins to become recruited to specific gene regulatory sequences. The association of TLE with numerous DNA-binding transcription factors is mediated by a defined site within the carboxyl-terminal WD40 repeat (WDR) domain of TLE and short Engrailed homology 1 (Eh1) or WRPW/Y sequences shared by many of their transcription partners [8]. Several Shh-regulated HD transcription factors, including Nkx2.2, Nkx6.1, Dbx1, and Dbx2, harbor Eh1 motifs and can physically interact with TLE [8]–[12]. Together, these observations suggest that TLE is involved in transcription repression mechanisms involved in ventral spinal cord patterning.

The aim of the present study was to determine whether TLE is important for the formation of the pMN/p3 boundary. This ventral boundary provides an ideal model system to study the involvement of TLE in spinal cord patterning because Nkx2.2, but not its cross-repressive partner Pax6, contains an Eh1 motif and can interact with TLE [10]. It is therefore expected that any *in vivo* perturbation of TLE function in the developing ventral spinal cord would affect the transcriptional repressor activity of Nkx2.2, but not Pax6. In contrast, at the p0/p1 and p1/p2 boundaries, both cross-repressive pairs of HD transcription factors (Dbx1/Nkx6.2 and Dbx2/Nkx6.1, respectively) contain Eh1 motifs and can bind TLE [10], a situation that would make the analysis of the consequences of TLE perturbation at these boundaries more complex. Here we describe the effects of either TLE overexpression or dominant-inhibition on the establishment of the pMN/p3 boundary in the developing spinal cord of chick embryos. Our results provide evidence that TLE acts to promote the formation of the p3 domain at the expense of the pMN domain by controlling the ventral extension of Pax6 expression, thereby favoring the differentiation of V3 INs.

## Materials and Methods

### Animals

All animal procedures were conducted in accordance with the guidelines of the Canadian Council for Animal Care and were approved by the Animal Care Committee of the Montreal Neurological Institute of McGill University (animal use protocol number 5468). Fertilized White Leghorn chicken eggs (Couvoir Simetin Hatchery, Mirabel, QC, Canada) were stored for a maximum of one week at 12°C and incubated at 37–38°C in a humidified 1550 Hatcher Incubator (GQF Manufacturing Company, Savannah, GA) until the required developmental stages. Chick embryos were staged according to Hamburger and Hamilton [13]. For staging of CD1 mouse embryos (Charles River, QC, Canada), the day of appearance of the vaginal plug was considered as embryonic day (E) 0.5.

### DNA Plasmids

The *pCMV2-FLAG-TLE1ΔQ* expression plasmid was generated by PCR amplification of the last 1884 bp of the 2312 bp coding sequence of full-length human *TLE1*, followed by subcloning into the EcoRV site of the *pCMV2-FLAG* vector. Plasmids *pCAG-EGFP*, *pCMV2-FLAG-TLE1*, *pFLAG-AES, pMyc-TLE4, pFOX-Ngn3promoter-luciferase, pCMV2-FLAG-Hes1*, and *pCMV2-FLAG-Hes1ΔWRPW* have been described previously [14]–[18]. In all FLAG epitope-tagged proteins, the FLAG epitope was at the amino terminus. The Myc epitope was also at the amino terminus in the Myc-TLE4 protein.

### *In Ovo* Electroporation

Chick spinal cord electroporation was performed at either HH stage 12–14 for subsequent immunohistochemical staining, or at HH stage 18–21 for coimmunoprecipitation and Western blotting. DNA was injected into the neural tube through a small eggshell window, under a Discovery.V8 stereomicroscope (Zeiss, Toronto, ON, Canada). The *pCMV2-FLAG* (control), *pCMV2-FLAG-TLE1, pCMV2-FLAG-TLE1ΔQ, pCMV2-FLAG-AES* and *pMyc-TLE4* constructs were all injected into the chick neural tube together with *pCAG-GFP* plasmid at a concentration ratio of 4∶1. The *pCMV2-FLAG-TLE1* and *pCMV2-FLAG-TLE1ΔQ* plasmids were used at the same concentration when injected together. The lower body of the chick embryos was then electroporated with a TSS20 Ovodyne Electroporator (Intracel, Shepreth, Royston, Hertfordshire, UK) with the following parameters: 20 V, 5 pulses 50 ms wide in a 1 s interval. Eggs were sealed with Parafilm and returned to the incubator until embryos were harvested at HH stage 26–28 for subsequent immunohistochemical staining, or at HH stage 22–24 for coimmunoprecipitation and Western blotting.

### Immunohistochemistry

Chick or mouse embryos were fixed in 4% paraformaldehyde for 1 h and cryoprotected in 30% sucrose. Cryoprotected embryos were embedded in Tissue-Tek Optimal Cutting Temperature (O.C.T.) compound (Sakura, Torrance, CA) and cryostat sections (14 µm) were subsequently prepared. Immunohistochemical staining was performed on sections from lumbar limb levels of electroporated chick embryos. Sections were washed with phosphate-buffered saline (PBS) for 5 min and non-specific staining was blocked with blocking solution containing 5% normal donkey serum and 0.1% Triton X-100 in PBS for 1 h. Subsequent incubations were performed with primary (2 h at room temperature or overnight at 4°C) and secondary (1 h) antibodies in blocking solution. The following primary antibodies were used: mouse anti-Nkx2.2 (Clone 74.5A5; 1∶50), mouse anti-Nkx6.1 (Clone F55A10; 1∶50), mouse anti-Hb9 (Clone 81.5C10; 1∶20), mouse anti-Isl1 (Clone 39.4D5; 1∶20) (Developmental Studies Hybridoma Bank, Iowa City, IA), mouse anti-Myc (1∶300; Abcam, Cambridge, MA), rabbit anti-Pax6 (1∶500; Covance, Emeryville, CA), rabbit anti-Olig2 (1∶500; Abcam, Cambridge, MA), rat anti-panTLE (1∶10) [14], [15], [19], rabbit anti-TLE1 (1∶500) [20], [21], or rabbit anti-TLE4 (1∶500) [21]. The fluorescent conjugated secondary antibodies used were the Alexa Fluor 488 and 555 series (1∶1,000; Molecular Probes, Invitrogen, Carlsbad, CA). Nuclei staining with Hoechst 33258 (1∶8,000; Invitrogen) was performed for 2 min. Slides were mounted with Fluoromount-G (SouthernBiotech, Birmingham, AL) and digital images were captured using Northern Eclipse software (Empix Imaging, Inc., Mississauga, ON, Canada) controlling a Digital Video Camera (DVC, Austin, TX) mounted on an Axioskop 2 fluorescence microscope (Zeiss, Toronto, ON, Canada).

To assess the effect of electroporation on the expression of specific cellular markers, the number of Hoechst stained nuclei positive for Nkx2.2, Pax6, Nkx6.1, Hb9 or Isl1, as well as GFP, were analyzed from ≥6 sections per embryo, n = 6 embryos per condition. Cell counts were performed using Image J software (National Institutes of Health, Bethesda, MD) and were expressed as the number of cells double positive for the relevant marker and GFP, as a percentage of total GFP+ cells (mean ± SEM). Data were analyzed using the unpaired *t*-test to compare 2 data sets or one-way ANOVA with Tukey's Multiple Comparison *post hoc* testing to compare more than 2 data sets (GraphPad Prism v.4.0). Values of *p*<0.05 were considered significant.

### Coimmunoprecipitation and Western Blotting

Lysates were prepared from electroporated chick spinal cord extracts and immunoprecipitation using either anti-FLAG (Sigma-Aldrich, Oakville, ON, Canada) or anti-Myc antibodies was performed as described [17], [18]. Immunoprecipitates and starting lysates were subjected to Western blotting analysis using anti-panTLE (1∶10) or anti-TLE1 (1∶1,000) antibodies. The anti-FLAG (1∶5,000), as well as anti-AES (1∶2,000; a kind gift from Dr. M.M. Taketo, Kyoto University, Kyoto, Japan), antibodies were used in Western blotting analyses to ensure the expression of exogenous proteins in the spinal cords of electroporated chick embryos.

### Transcription Assays

HEK293 cells were obtained from the American Tissue Type Collection (Manassas, VA) [14], [15] and transiently transfected using SuperFect reagent according to the manufacturers' protocol (Qiagen, Mississauga, ON, Canada). Transfections were performed with the reporter construct *pFOX-Ngn3promoter-luciferase* (1 µg/transfection) alone or with *pCMV2-FLAG-Hes1* or *pCMV2-FLAG-Hes1ΔWRPW* (0.1 µg/transfection), in the absence or presence of *pCMV2-FLAG-TLE1* or *pCMV2-FLAG-TLE1ΔQ* (0.2 µg/transfection). In each case, *pCMV-βgal* (0.5 µg/transfection) was used to normalize for transfection efficiency and the total amount of transfected DNA was adjusted to 2.5 µg per well using *pcDNA3*. Luciferase activity was determined 24 h after transfection and results are expressed as mean ± SEM.

## Results

### TLE expression in the developing ventral spinal cord

Transcriptional corepressors of the TLE family are broadly expressed throughout development [8]. Because the spatiotemporal pattern of TLE protein expression in the developing vertebrate spinal cord had not been characterized, we subjected sections from the spinal cord of developing chick and mouse embryos to immunohistochemical analysis using a previously validated ‘panTLE’ monoclonal antibody that specifically recognizes all TLE proteins from invertebrates to humans. At the same time, we compared TLE expression to that of the following ventral spinal progenitor domain markers: Pax6 (p0-pMN domains), Nkx6.1 (p2–p3 domains), Olig2 (pMN domain) and Nkx2.2 (p3 domain) [1], [2] (Fig. 1A). TLE immunoreactivity was detected in the ventral neural tube of chick embryos as early as HH stage 12–14 and persisted throughout the period of motor neurogenesis. At HH stage 18–20, many TLE-immunoreactive cells were observed throughout the dorsoventral axis (Fig. 1B–E), consistent with the broad expression of these proteins within and outside of the nervous system [8]. TLE immunoreactivity overlapped with the expression of Pax6, Olig2, Nkx6.1, and Nkx2.2 (Fig. 1B–E). Specifically, significant overlap was observed between TLE expression and high Pax6 immunoreactivity, dorsal to the Olig2+ domain, suggesting expression of TLE proteins in p0–p2 progenitors (Fig. 1B). TLE-expressing cells were also present in the domain dorsal to Nkx2.2 immunoreactivity and characterized by the expression of low levels of Pax6 (Fig. 1B), as well as by the expression of Olig2 (Fig. 1C) and Nkx6.1 (Fig. 1D). This observation suggests that TLE proteins are also expressed in the pMN progenitor domain. Finally, virtually all of the most ventral cells expressing Nkx6.1 and Nkx2.2 were positive for TLE immunoreactivity (Fig. 1D and 1E), indicating that TLE proteins are expressed in p3 progenitors. A similar pattern of expression was observed in the mouse spinal cord at the approximately equivalent developmental stage of E10.5 (supporting information Fig. S1). We also observed a similar expression pattern when previously characterized antibodies against TLE1 or TLE4 were used, confirming the results obtained with the panTLE antibody and demonstrating expression of these two TLE family members in the developing spinal cord (Fig. S2). Together, these results show that TLE proteins are coexpressed together with HD transcription factors that pattern the ventral spinal cord.

### Increased numbers of Nkx2.2-positive progenitors and V3 interneurons following forced expression of TLE1 in the developing chick spinal cord

**Figure 2:**
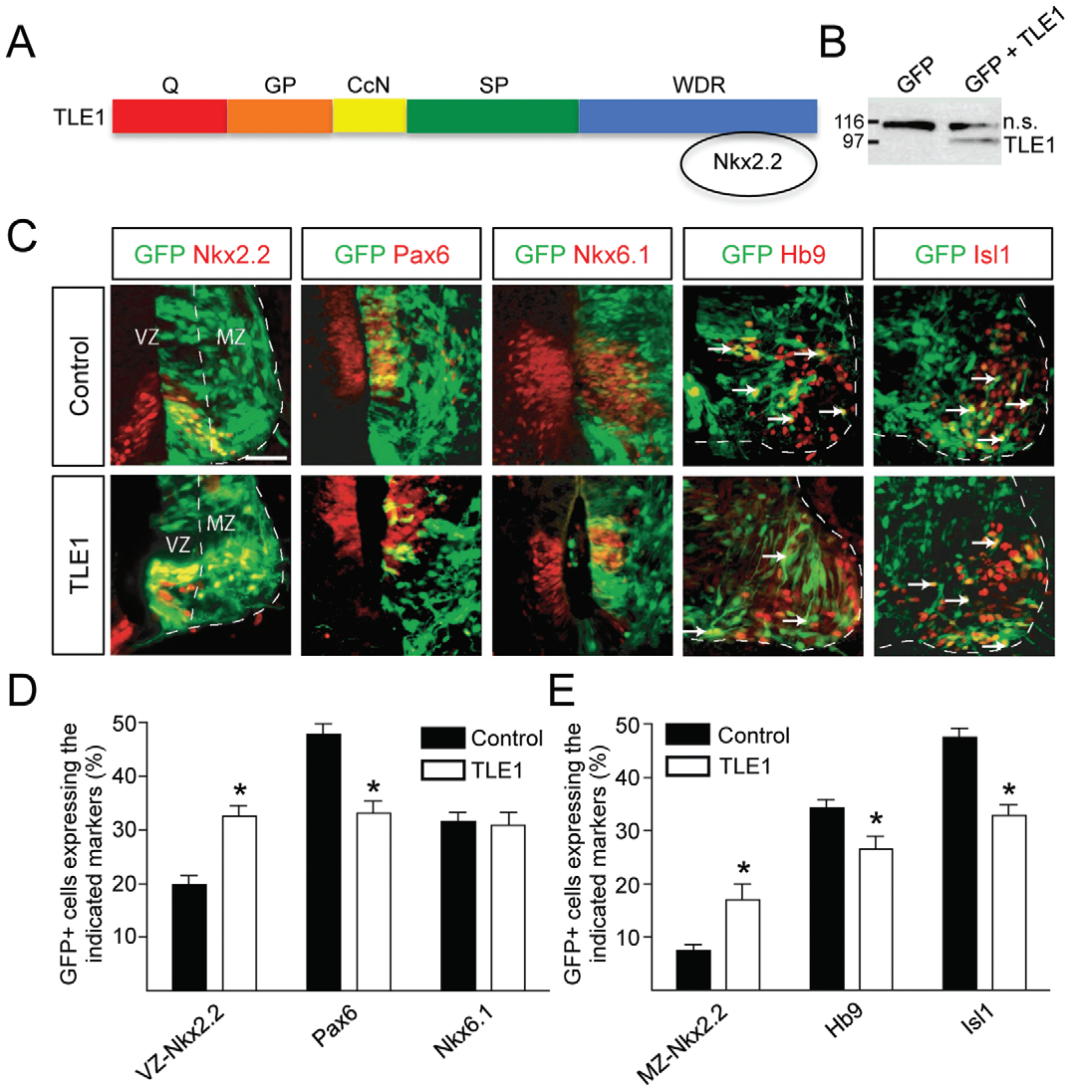
Effect of TLE1 overexpression in the developing chick ventral spinal cord. (A) Schematic representation of the TLE domain structure. Notice the Q domain involved in oligomerization and transcriptional repression and the WDR domain important for protein-protein interactions [8]. Nkx2.2 binds to the TLE WDR domain using an Eh-1 motif. (B) Western blotting analysis of lysates from chick embryo spinal cords electroporated with plasmids encoding GFP alone or together with FLAG epitope-tagged TLE1 demonstrating the expression of exogenous TLE1 using anti-FLAG antibody. “n.s.” indicates a non-specific band detected by this antibody. (C) Double-labeling analysis of the expression of GFP and the indicated proteins in embryos electroporated with GFP alone (control) or GFP+TLE1 (TLE1). Nkx2.2+ cells were observed in both the ventricular zone (VZ) and mantle zone (MZ). Arrows in the two right-hand columns point to examples of double-labeled cells coexpressing GFP and either Hb9 or Isl1. (D and E) Quantification of the numbers of electroporated cells (GFP+) expressing Nkx2.2 [in either the VZ (D) or the MZ (E)], Pax6, Nkx6.1, Hb9, or Isl1, as indicated. TLE1 overexpression caused an increase in the number of Nkx2.2+ cells in the VZ, with a concomitant decrease in Pax6+ cells. The number of cells expressing Nkx6.1 was not altered. These changes were associated with an increase in Nkx2.2+ cells in the MZ and a decrease in the number of electroporated cells expressing the MN markers Hb9 and Isl1. Data are expressed as mean ± SEM (*p<0.05). Scale bars = 50 µm.

The coexpression of TLE and Nkx2.2, together with the demonstration that these proteins can form a complex [10], [11], suggests that TLE may functionally interact with Nkx2.2 during spinal cord patterning. To examine this possibility, we performed *in ovo* electroporation experiments to overexpress TLE proteins in the ventral spinal cord of developing chick embryos. This approach was based on the hypothesis that TLE overexpression might enhance the transcription repression activity of Nkx2.2 without affecting the transcription repression activity of Pax6, which is not endowed with an Eh1 motif and does not bind to TLE [10], thereby possibly causing a perturbation of the mutual cross-repression between Nkx2.2 and Pax6 during the establishment of the pMN/p3 boundary. HH stage 12–14 embryos were electroporated with a plasmid encoding GFP, to mark electroporated cells, alone or in combination with plasmids encoding full-length TLE1 or TLE4 tagged with either a FLAG or Myc epitope, respectively. Double-labeling experiments revealed that virtually every GFP-expressing cell coexpressed Myc-tagged TLE4 in the ventral spinal cord of electroporated embryos both 48 and 72 h after electroporation (Fig. S3). Although similar studies using anti-FLAG epitope antibodies were not technically possible due to background cross-reactivity on chick tissues, Western blotting analysis of electroporated embryos confirmed the expression of exogenous TLE1 (Fig. 2B). Because the plasmid driving expression of TLE1 resulted in a more robust exogenous TLE1 expression than the one encoding TLE4, subsequent studies were conducted using the TLE1-expression plasmid.

We observed that overexpression of TLE1 caused a significant decrease in the number of progenitor cells that expressed Pax6 in the germinative zone [‘ventricular zone’ (VZ), which contains undifferentiated neural progenitor cells], compared to the control condition (Fig. 2C and 2D). This situation was correlated with a parallel increase of electroporated cells expressing Nkx2.2 in the VZ (‘VZ Nkx2.2+ cells’, which define the p3 domain) (Fig. 2C and 2D). The number of Nkx6.1+ cells, which are found throughout the p2–p3 domains, was unchanged when TLE1 was overexpressed (Fig. 2D), indicating that the effect of exogenous TLE1 expression on the number of Pax6+ and Nkx2.2+ cells was specific. These results suggest that TLE1 overerexpression in the ventral neural tube leads to enhanced Nkx2.2-mediated transcriptional repression of *Pax6*. Further, they suggest that the ensuing reduction in Pax6 expression at the pMN/p3 boundary results in a dorsal derepression of *Nkx2.2* expression and increased numbers of Nkx2.2+ cells.

We next examined if the perturbation of Nkx2.2 and Pax6 expression caused by exogenous TLE1 expression in progenitor cells was correlated with alterations in postmitotic neuron numbers in the mantle zone (MZ). During spinal cord development, the p3 domain gives rise to V3 INs, which maintain Nkx2.2 expression transiently as they become postmitotic and migrate away from the progenitor zone into the MZ. The pMN domain generates MNs that express proteins, such as Hb9 and Isl1, which are not present in ventral INs [22]–[25]. Our studies revealed a significant increase in Nkx2.2+ cells located in the MZ (‘MZ Nkx2.2+ cells’), most likely corresponding to differentiating/ed V3 INs (Fig. 2C and 2E). This effect was correlated with a significant decrease in the number of cells expressing the MN markers Hb9 and Isl1 (Fig. 2C and 2E). Taken together, these findings provide evidence that TLE overexpression alters the establishment of the correct number of progenitor cells expressing either Pax6 or Nkx2.2 at the pMN/p3 boundary by promoting the formation of supernumerary p3 progenitors at the expense of pMN progenitors. In turn, this situation is correlated with the generation of supernumerary V3 INs at the expense of postmitotic MNs.

### Increased numbers of Pax6-positive progenitors and postmitotic motor neurons following dominant negative inhibition of TLE in the developing chick spinal cord

**Figure 3:**
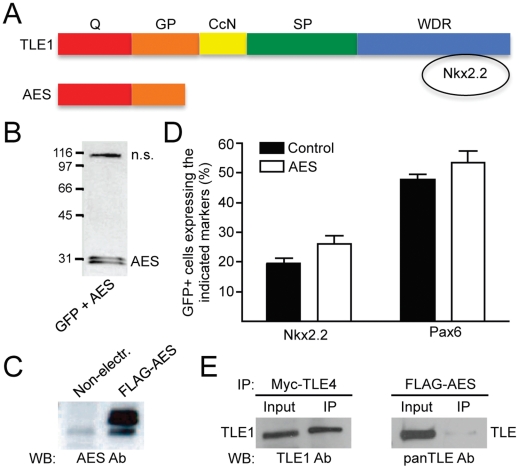
Lack of effect of AES overexpression on ventral spinal cord Pax6+ and Nkx2.2+ progenitor populations. (A) Schematic comparison of the structure of AES to that of full-length TLE. AES lacks the WDR domain involved in Nkx2.2 binding but retains the amino-terminal Q domain [8]. (B and C) Western blotting analysis of lysates from chick embryo spinal cords electroporated with plasmids encoding GFP together with FLAG epitope-tagged AES using either anti-FLAG (B) or anti-AES (C) antibodies. (B) “n.s.” indicates a non-specific band detected by the anti-FLAG antibody. (C) Exogenous AES was dramatically overexpressed in electroporated spinal cords. (D) Quantification of the number of GFP+ cells expressing Nkx2.2 or Pax6 in chick embryos electroporated with GFP alone (Control) or together with AES (AES). AES had no significant effect on the number of either Nkx2.2+ or Pax6+ progenitor cells. (E) Coimmunoprecipitation experiments performed using lysates from chick embryo spinal cords electroporated with plasmids encoding either Myc-tagged TLE4 or FLAG-tagged AES, as indicated. TLE4 or AES were immunoprecipitated (IP) using anti-Myc or anti-FLAG antibodies, respectively, followed by Western blotting (WB) analysis of input lysate (10%) and immunoprecipitated material using an anti-TLE1 antibody that does not cross-react with TLE4 or a panTLE antibody that recognizes all full-length TLE proteins because it is directed against the WDR domain [19]. Endogenous TLE1 coimmunoprecipitated efficiently with exogenous TLE4. In contrast, only a modest coimmunoprecipitation of AES with endogenous TLE was detected.

To complement the analysis based on TLE overexpression, we investigated the effect of inhibiting TLE function during ventral spinal cord patterning. RNA interference strategies targeting *TLE* transcripts are technically challenging in the context of the developing spinal cord due to the fact that several *TLE* genes are expressed in this tissue (Fig. S2 and Ref. [10]). This situation would require a simultaneous knockdown of multiple TLE family members. We therefore opted to employ dominant negative approaches that would target all TLE proteins concurrently. One approach was based on the previous suggestion that a Groucho/TLE-related protein termed amino-terminal enhancer of split (AES) or Grg5 (hereafter referred to as AES) [8], [16] could exert a dominant-negative effect on endogenous TLE in the developing spinal cord [10]. AES is a short protein that shares significant sequence homology with the N-terminal Q domain found in all full-length TLE proteins. However, AES completely lacks the WDR domain that enables TLE to interact with transcription factors harboring Eh1 or WRPW/Y motifs [8] (Fig. 3A). Moreover, AES is believed to lack transcriptional corepresor activity [8]. Because of these features and the fact that AES is theoretically competent to form hetero-oligomers with TLE [26], AES is believed to be able to act as a dominant-negative inhibitor of the transcriptional corepressor functions of TLE [10], although this possibility has been called into question by several studies [27]–[29].

HH stage 12–14 chick embryos were electroporated with a plasmid encoding a FLAG-tagged form of AES that was shown to be biologically competent based on its ability to bind to the NFκB subunit, RelA, like full-length TLE [16]. *In ovo AES* electroporation resulted in exogenous AES protein expression at levels that were significantly higher than those of endogenous AES and thus presumably sufficient to achieve dominant inhibition of TLE *in vivo* (Fig. 3B and 3C). Despite this level of overexpression, however, exogenous AES had no significant effect on the number of either Nkx2.2+ or Pax6+ progenitor cells (Fig. 3D). This situation was correlated with the demonstration that exogenous AES associated only modestly with endogenous TLE in the spinal cord, in contrast to the efficient interaction of endogenous TLE with electroporated TLE4 (Fig. 3E). These results provide evidence that exogenous expression of AES does not have a detectable effect on the expression of Pax6 and Nkx2.2 at the pMN/p3 boundary, likely because AES does not exert a dominant-inhibitory effect on endogenous TLE in this context.

**Figure 4:**
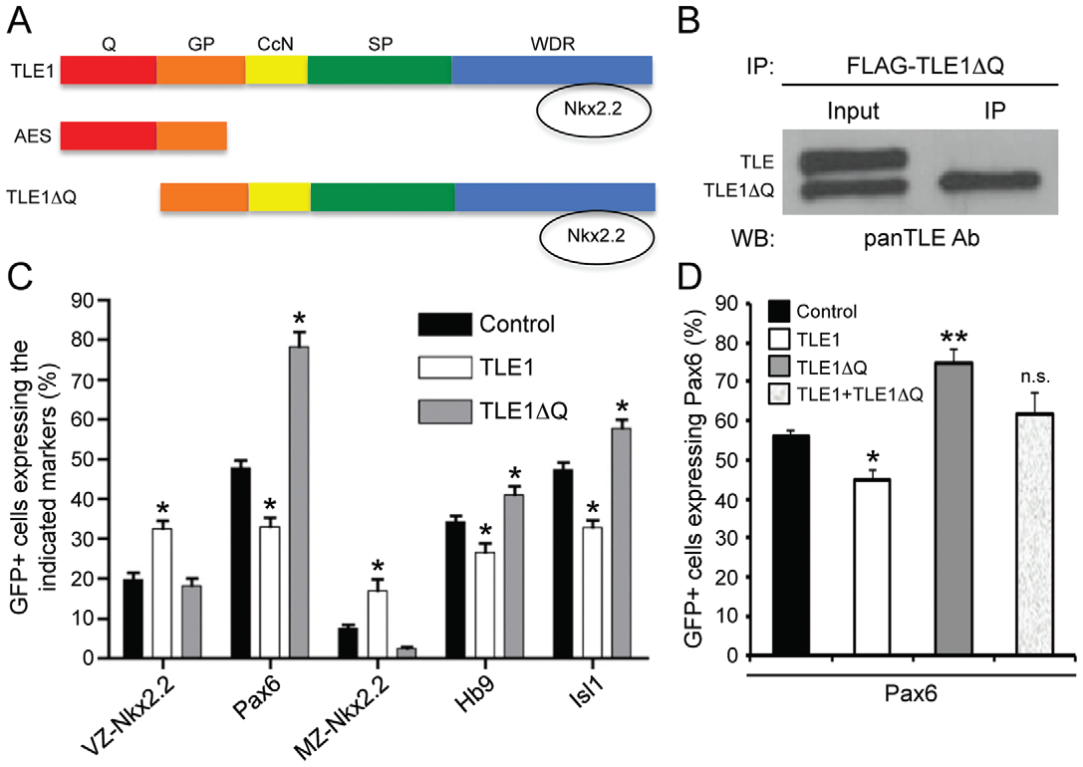
Effect of TLE1ΔQ expression on ventral spinal cord Pax6+ and Nkx2.2+ progenitor populations and neuronal fate acquisition. (A) Schematic representation of TLE1ΔQ, compared to TLE1 and AES, depicting the lack of the Q domain but retention of the WDR domain in TLE1ΔQ. (B) Coimmunoprecipitation experiments performed using lysates from chick embryo spinal cords electroporated with plasmid encoding FLAG-TLE1ΔQ. Immunoprecipitation (IP) was performed using anti-FLAG antibody, followed by Western blotting (WB) analysis of input lysate (10%) and immunoprecipitated material using a panTLE antibody that recognizes all full-length TLE proteins and also TLE1ΔQ because it is directed against the WDR domain [19]. Endogenous TLE did not coimmunoprecipitate with exogenous TLE1ΔQ. (C) Quantification of the number of GFP+ cells expressing Nkx2.2 [in either the ventricular zone (VZ) or marginal zone (MZ)], Pax6, Hb9, or Isl1 in chick embryos electroporated with GFP alone or together with TLE1 or TLE1ΔQ. Expression of TLE1ΔQ resulted in an increase in the number of Pax6+ progenitor cells as well as Hb9+ and Isl1+ MNs compared to the control conditions. These effects were opposite to the effects of TLE1. See Figure S5 for double-labeling immunohistochemical analysis of electroporated embryos. (D) Quantification of the number of GFP+ cells expressing Pax6 in chick embryo spinal cord electroporated with GFP alone or together with TLE1, TLE1ΔQ, or TLE1 and TLE1ΔQ together, as indicated. Data in (C and D) are expressed as mean ± SEM (*p<0.05; **p<0.01; n.s., not significant).

Due to these results, we adopted a different approach to inhibit endogenous TLE function based on the use of an engineered mutant form of TLE1 (‘TLE1ΔQ’), which lacks the Q domain necessary for TLE oligomerization and transcriptional repression [30] but retains all other TLE domains (Fig. 4A). TLE1ΔQ is predicted to act as a dominant-inhibitor of endogenous TLE because it harbors the WDR domain that mediates interaction with many TLE-binding proteins but lacks the Q domain required for transcriptional repression. As a result, TLE1ΔQ should be able to ‘titrate’ away endogenous TLE-binding proteins if expressed at sufficiently high levels, without providing a transcriptional corepression activity. In agreement with this possibility, we showed that TLE1ΔQ displayed a dominant-inhibitory effect on the ability of endogenous TLE to act as transcriptional corepressor for the bHLH protein Hes1, which binds to the WDR domain of TLE using a WRPW motif (Fig. S4).

We therefore examined the effect of expressing TLE1ΔQ in the developing chick spinal cord by *in ovo* electroporation. Coimmunoprecipitation studies showed that TLE1ΔQ did not interact with endogenous full-length TLE proteins (Fig. 4B). More importantly, expression of TLE1ΔQ led to increased numbers of Pax6+ cells in the ventral spinal cord, in contrast to the decrease in Pax6-expressig cells caused by TLE1 (Fig. 4C and Fig. S5). The effect of TLE1ΔQ on Pax6 expression was blocked by the coexpression of TLE1, demonstrating that these proteins can act in an antagonistic manner and consistent with a dominant-negative function of TLE1ΔQ (Fig. 4D). The number of Nkx2.2+ cells was not altered upon electroporation of TLE1ΔQ compared to the control conditions. The increase in Pax6+ progenitors was correlated with an increase in Hb9+ and Isl1+ cells in the MZ, contrary to the decrease in the number of cells expressing these MN markers caused by TLE1 (Fig. 4C). Together, these results suggest that TLE1ΔQ exerts a dominant-negative effect on endogenous TLE when expressed in the ventral spinal cord. More importantly, the combined results of electroporation experiments using TLE1 and TLE1ΔQ provide evidence that endogenous TLE is important for the establishment of the correct number of progenitor cells expressing either Pax6 or Nkx2.2 at the pMN/p3 boundary during ventral spinal cord patterning.

## Discussion

**Figure 5:**
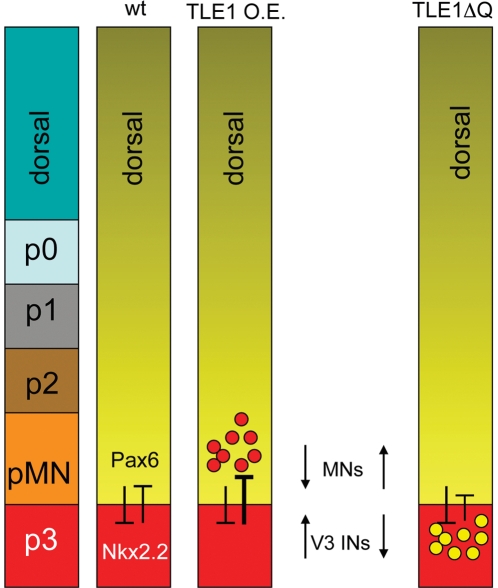
Schematic summary of the effects of TLE perturbations on Pax6+ and Nkx2.2+ ventral progenitor populations and neuronal fate acquisition. Pax6 and Nkx2.2 normally repress the expression of each other to establish the pMN/p3 boundary. Overexpression (O.E.) of TLE1 increases the number of Nkx2.2+ progenitor cells and V3 INs at the expense of Pax6+ progenitor cells and somatic MNs. Conversely, exogenous expression of TLE1ΔQ results in an increase in Pax6+ progenitor cells and MNs.

The present study has provided evidence that TLE proteins are expressed throughout the dorsoventral axis of the developing chick and mouse spinal cord, including within the pMN and p3 progenitor domains. Overexpression of TLE1 in the developing chick ventral spinal cord results in an increase in Nkx2.2+ p3 progenitor cells at the expense of Pax6+ pMN progenitor cells. This perturbation is correlated with an increase in V3 INs and an attendant decrease in postmitotic MNs in the MZ (summarized in Fig. 5). Conversely, forced expression of TLE1ΔQ, a proven dominant-negative inhibitor of the corepressor function provided by TLE to proteins that, like Nkx2.2, bind to TLE via the WDR domain, results in an increase in both Pax6+ pMN progenitors and postmitotic MNs (Fig. 5). These findings support the view that the establishment of the correct number of p3 and pMN progenitor cells in the ventral spinal cord is dependent on transcriptional repression mediated by TLE.

Based on the coexpression of TLE and Nkx2.2 in the ventral spinal cord (this study) and the demonstrated physical interaction of these proteins [10], [11], we propose that TLE acts as a transcriptional corepressor with Nkx2.2 to repress *Pax6* expression in the p3 domain and ultimately promote the V3 IN fate. This possibility is in agreement with the observation that the effect of exogenous TLE1 expression is similar to the previously described effect of exogenous Nkx2.2 expression, namely suppression of Pax6 expression and MN fate and promotion of the V3 IN fate [1], [2], [31], [32]. We recognize that we cannot exclude the possibility that the effects caused by TLE1 overexpression might have been due to the recruitment of TLE1 by transcription factors alternative, or in addition, to Nkx2.2. For instance, Nkx2.9 is transiently expressed in the p3 domain of embryonic mice and can also interact with TLE [10]. Moreover, Nkx2.2 and Nkx2.9 appear to have redundant activities [1], [10], [31]. However, the expression of Nkx2.9 in the p3 domain is almost extinguished by E10.5 in the mouse, suggesting that at the stage when our experiments were performed in the chick, the expression of Nkx2.9 in this domain might not have been an important factor. Our results showed further that dominant inhibition of TLE did not cause a detectable change in the number of Nkx2.2+ cells. This observation is consistent with the notion that interfering with TLE activity does not affect Pax6 function, likely because TLE does not work together with Pax6. This possibility is in agreement with the fact that Pax6 does not contain a TLE-binding motif, contrary to Nkx2.2.

A role for TLE in ventral neural patterning mediated by HD transcription factors such as Nkx2.2 has previously been suggested [10]. In agreement with the results presented herein at the protein level, Muhr and colleagues [10] demonstrated the expression of *TLE* mRNA in the developing chick and mouse ventral spinal cord. However, contrary to our present findings, they concluded that TLE inhibits the V3 IN fate based on the observation that ectopic expression of AES in the chick ventral neural tube resulted in ectopic expression of Nkx2.2 in cells located dorsal to the p3 domain. The conclusion of Muhr and colleagues [10] was based on the assumption that AES was a *bona fide* dominant negative inhibitor of all TLE functions. Although there is evidence that AES may have dominant-inhibitory effects on those TLE functions that involve recruitment of DNA-binding proteins via the Q domain [8], [9], several studies have called into question the general validity of this postulate, especially with regard to those TLE functions involving proteins that bind to the TLE WDR domain. For instance, the overexpression of AES in developing medaka fish was shown to cause biological effects that were in some cases opposite to, and in other case the same as, those caused by expression of TLE [27]. Moreover, studies in *C. elegans* have identified LSY-22 as an ortholog of vertebrate AES and shown that loss-of-function alleles of *lsy-22* and *unc-37*, the *C. elegans TLE* ortholog, display identical phenotypes in neuronal fate specification and in other developmental contexts, suggesting that AES-like proteins may promote TLE function in specific contexts, rather than acting as dominant-negative regulators [29]. These genetic observations are consistent with previous studies showing that AES does not act as a negative regulator of the transcriptional corepressor effect of TLE on Hes1, another protein that, like Nkx2.2, binds to the TLE WDR domain [33].

On the basis of these observations, it is plausible that the dorsal expansion of the Nkx2.2+ domain observed by Muhr and colleagues [10] upon electroporation of AES was due to the overexpression of AES mimicking, rather than inhibiting, the effect of endogenous TLE, similar to the situation observed in gain-of-function studies in medaka fish [27]. This conclusion is also supported by the present demonstration that expression of TLE1ΔQ, a validated TLE dominant negative form in the context of proteins that bind to the TLE WDR domain, caused increased numbers of Pax6+ progenitors and postmitotic MNs in the ventral spinal cord, opposite to the effect of TLE1. This latter finding also suggests that it is highly unlikely that the effects of exogenous TLE1 expression might have resulted from a dominant negative effect caused by the sequestration of other transcriptional corepressors by exogenous TLE1. This is also suggested by the similarity of the results of the overexpression of TLE1 with those obtained after overexpression of Nkx2.*2* [31], [32].

In summary, the results of the present study demonstrate a role for TLE transcriptional corepressors in the establishment of the correct number of p3 and pMN progenitor cells in the ventral spinal cord and in the promotion of the V3 IN fate. As has been discussed previously [8], [28], [29], this study also provides further evidence that AES is not a general dominant-inhibitor of TLE, highlighting the need for caution when interpreting the results of studies based on the use of AES overexpression strategies.

## Supporting Information

Figure S1 TLE expression in the ventral spinal cord of E10.5 mouse embryos. Horizontal sections through the spinal cord of E10.5 mouse embryos were subjected to double-labeling immunofluorescence analysis of the expression of TLE and either Pax6 (A), Nkx6.1 (B), Olig2 (C), or Nkx2.2 (D). A panTLE antibody was used in each case. TLE expression was particularly evident in the region of Nkx6.1 expression (p2–p3), which included both Olig2 and Nkx2.2 expression domains. Scale bar: 100 µm.
(TIF)

Figure S2 TLE1 and TLE4 expression in the ventral spinal cord of E10.5 mouse embryos. Horizontal sections through the spinal cord of E10.5 mouse embryos were subjected to double-labeling immunofluorescence analysis of the expression of TLE1 (A) or TLE4 (B) together with a panTLE antibody, as indicated. ‘Hoe’, Hoechst staining.
(TIF)

Figure S3 Coexpression of GFP and TLE in electroporated chick embryo spinal cord. Double-labeling analysis of the expression of GFP and Myc-tagged TLE4 (using an anti-Myc antibody) in the ventral spinal cord of electroporated chick embryos 48 h (A–D) or 72 h (E–H) after electroporation. Boxes in panels (A) and (E) demarcate areas shown at higher magnification in panels (B–D) and (F–H), respectively ‘Hoe’, Hoechst staining. Virtually all GFP-expressing cells also express Myc-tagged TLE4.
(TIF)

Figure S4 Dominant negative effect of TLE1ΔQ on endogenous TLE. (A) Transient transfection-transcription assays. HEK293 cells were transfected with a reporter plasmid encoding luciferase under the control of the *Ngn3* promoter, which contains multiple Hes1 binding sites (Promoter). This vector was transfected alone (luciferase activity considered as 100%) or together with a Hes1-expression plasmid to measure transcriptional repression (second bar). Coexpression of TLE1 resulted in enhanced repression (third bar); in contrast, coexpression of TLE1ΔQ caused derepression of reporter gene expression above basal levels (fourth bar), most likely due to the fact that HEK293 cells endogenously express TLE and Hes1 [17], [32]. A mutated form of Hes1 lacking the WRPW motif that mediates TLE binding (Hes1ΔWRPW) was unable to repress transcription and instead caused reporter gene derepression, most likely by acting as a dominant negative inhibitor of endogenous Hes1 (fifth bar). This effect was not influenced by TLE1ΔQ. (A) Western blotting analysis using anti-FLAG antibody confirmed the expression of exogenous TLE1, TLE1ΔQ and Hes1 proteins in these transcription assays.
(TIF)

Figure S5 Double-labeling analysis of the expression of GFP and either Nkx2.2 (left column), Isl1 (middle column) or HB9 (right column) in the ventral spinal cord of chick embryos electroporated with GFP alone or together with TLE1 or TLE1ΔQ, as indicated. ‘Hoe’, Hoechst staining.
(TIF)
