# Supplementary figures and images for: Establishment of Motor Neuron-V3 Interneuron Progenitor Domain Boundary in Ventral Spinal Cord Requires Groucho-Mediated Transcriptional Corepression
Source: PLoS One. 2012 Feb 17;7(2):e31176. doi: 10.1371/journal.pone.0031176 (PMC3281934; doi:10.1371/journal.pone.0031176)

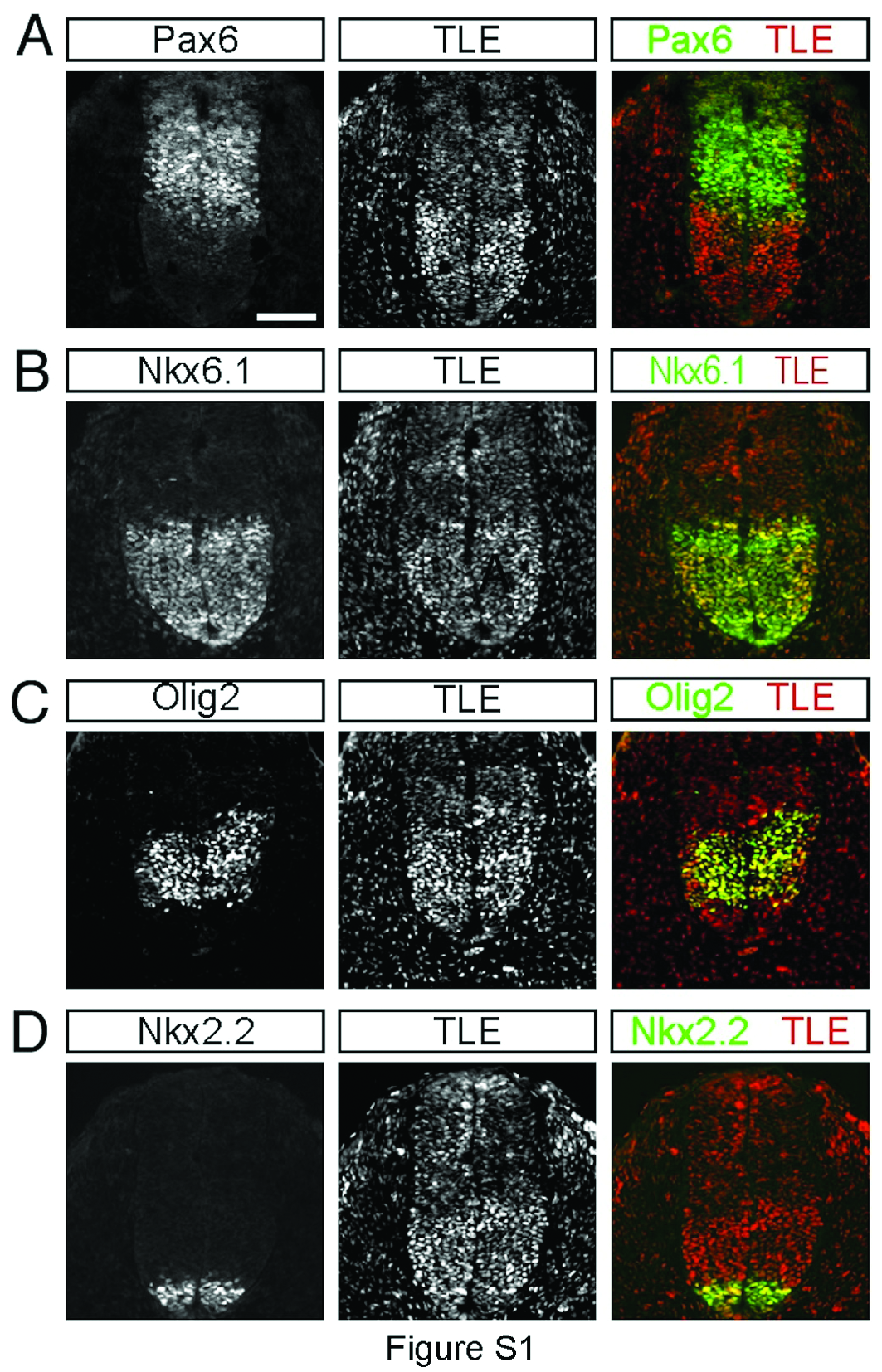

Supplement: Figure S1 — TLE expression in the ventral spinal cord of E10.5 mouse embryos. Horizontal sections through the spinal cord of E10.5 mouse embryos were subjected to double-labeling immunofluorescence analysis of the expression of TLE and either Pax6 (A), Nkx6.1 (B), Olig2 (C), or Nkx2.2 (D). A panTLE antibody was used in each case. TLE expression was particularly evident in the region of Nkx6.1 expression (p2–p3), which included both Olig2 and Nkx2.2 expression domains. Scale bar: 100 µm. (TIF) [file pone.0031176.s001.tif]

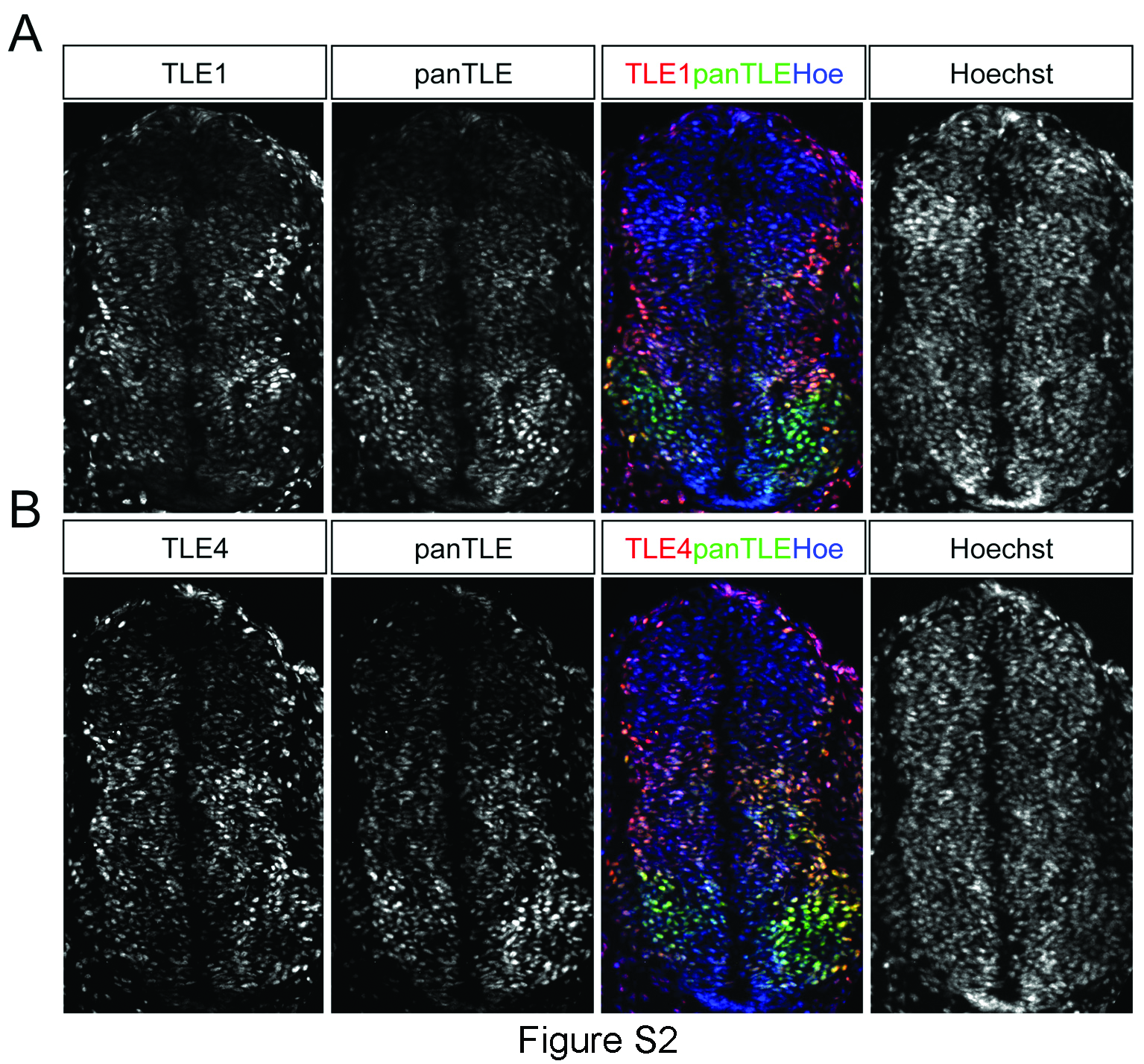

Supplement: Figure S2 — TLE1 and TLE4 expression in the ventral spinal cord of E10.5 mouse embryos. Horizontal sections through the spinal cord of E10.5 mouse embryos were subjected to double-labeling immunofluorescence analysis of the expression of TLE1 (A) or TLE4 (B) together with a panTLE antibody, as indicated. ‘Hoe’, Hoechst staining. (TIF) [file pone.0031176.s002.tif]

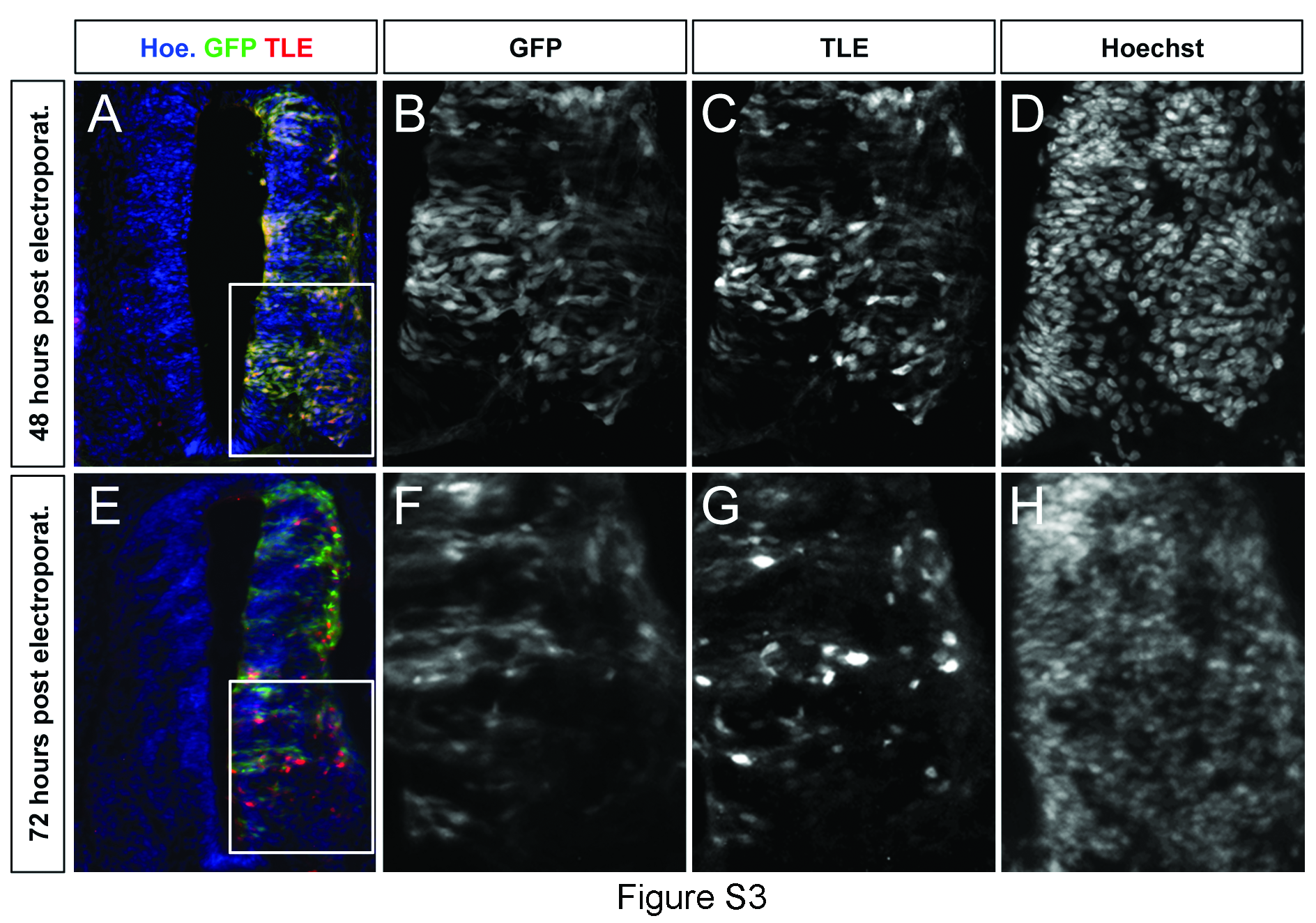

Supplement: Figure S3 — Coexpression of GFP and TLE in electroporated chick embryo spinal cord. Double-labeling analysis of the expression of GFP and Myc-tagged TLE4 (using an anti-Myc antibody) in the ventral spinal cord of electroporated chick embryos 48 h (A–D) or 72 h (E–H) after electroporation. Boxes in panels (A) and (E) demarcate areas shown at higher magnification in panels (B–D) and (F–H), respectively ‘Hoe’, Hoechst staining. Virtually all GFP-expressing cells also express Myc-tagged TLE4. (TIF) [file pone.0031176.s003.tif]

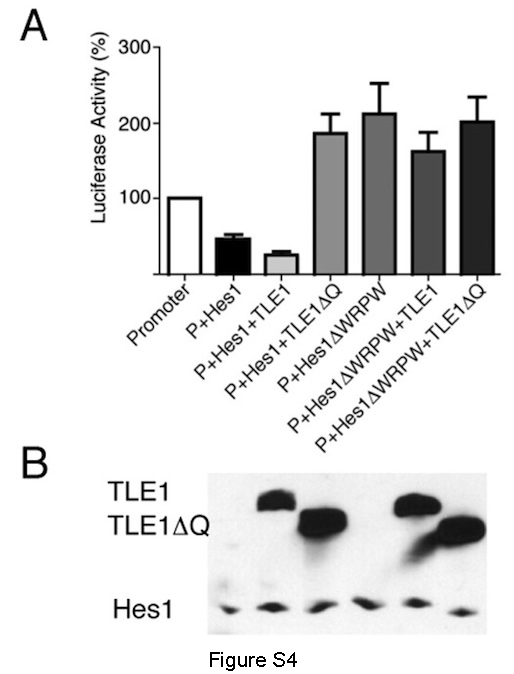

Supplement: Figure S4 — Dominant negative effect of TLE1ΔQ on endogenous TLE. (A) Transient transfection-transcription assays. HEK293 cells were transfected with a reporter plasmid encoding luciferase under the control of the Ngn3 promoter, which contains multiple Hes1 binding sites (Promoter). This vector was transfected alone (luciferase activity considered as 100%) or together with a Hes1-expression plasmid to measure transcriptional repression (second bar). Coexpression of TLE1 resulted in enhanced repression (third bar); in contrast, coexpression of TLE1ΔQ caused derepression of reporter gene expression above basal levels (fourth bar), most likely due to the fact that HEK293 cells endogenously express TLE and Hes1 [17], [32]. A mutated form of Hes1 lacking the WRPW motif that mediates TLE binding (Hes1ΔWRPW) was unable to repress transcription and instead caused reporter gene derepression, most likely by acting as a dominant negative inhibitor of endogenous Hes1 (fifth bar). This effect was not influenced by TLE1ΔQ. (A) Western blotting analysis using anti-FLAG antibody confirmed the expression of exogenous TLE1, TLE1ΔQ and Hes1 proteins in these transcription assays. (TIF) [file pone.0031176.s004.tif]

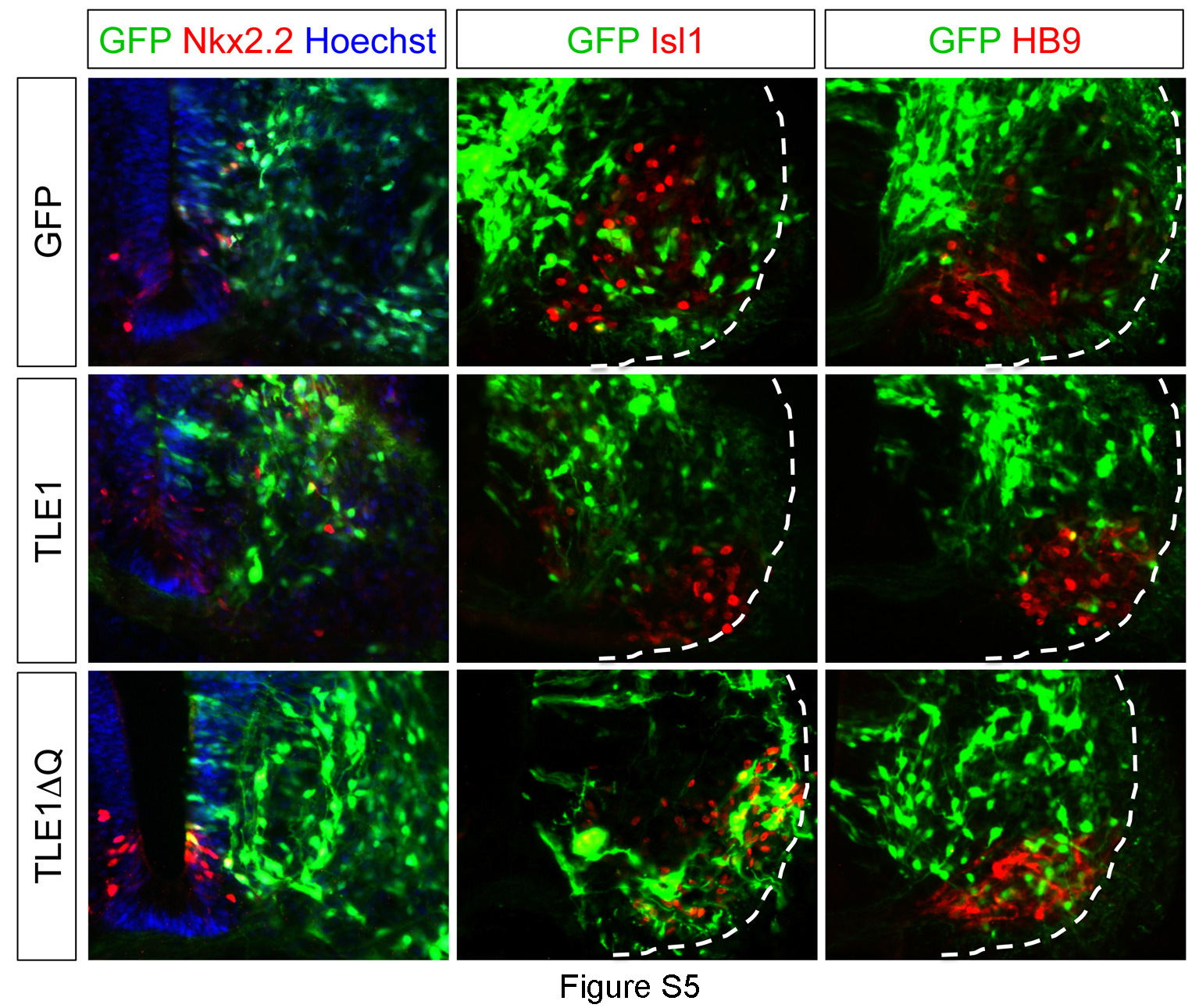

Supplement: Figure S5 — Double-labeling analysis of the expression of GFP and either Nkx2.2 (left column), Isl1 (middle column) or HB9 (right column) in the ventral spinal cord of chick embryos electroporated with GFP alone or together with TLE1 or TLE1ΔQ, as indicated. ‘Hoe’, Hoechst staining. (TIF) [file pone.0031176.s005.tif]
